# Intellectual contributions meriting authorship: Survey results from the top cited authors across all science categories

**DOI:** 10.1371/journal.pone.0198117

**Published:** 2019-01-16

**Authors:** Gregory S. Patience, Federico Galli, Paul A. Patience, Daria C. Boffito

**Affiliations:** 1 Department of Chemical Engineering, Polytechnique Montréal, Montréal, QC, Canada; 2 Department of Electrical Engineering, Polytechnique Montréal, Montréal, QC, Canada; Indiana University Bloomington, UNITED STATES

## Abstract

Authorship is the currency of an academic career for which the number of papers researchers publish demonstrates creativity, productivity, and impact. To discourage coercive authorship practices and inflated publication records, journals require authors to affirm and detail their intellectual contributions but this strategy has been unsuccessful as authorship lists continue to grow. Here, we surveyed close to 6000 of the top cited authors in all science categories with a list of 25 research activities that we adapted from the National Institutes of Health (NIH) authorship guidelines. Responses varied widely from individuals in the same discipline, same level of experience, and same geographic region. Most researchers agreed with the NIH criteria and grant authorship to individuals who draft the manuscript, analyze and interpret data, and propose ideas. However, thousands of the researchers also value supervision and contributing comments to the manuscript, whereas the NIH recommends discounting these activities when attributing authorship. People value the minutiae of research beyond writing and data reduction: researchers in the humanities value it less than those in pure and applied sciences; individuals from Far East Asia and Middle East and Northern Africa value these activities more than anglophones and northern Europeans. While developing national and international collaborations, researchers must recognize differences in peoples values while assigning authorship.

## Introduction

The scientific process requires ingenuity and individuals that contribute creativity to answering the research question merit authorship [1, 2]. However authorship lists continue to climb [3, 4] despite the widespread dissemination of guidelines to dissuade ambiguous attribution [5, 6]: in 2013, articles averaged 5 to 10 authors while in 1993 there were only 2 to 3 [7]. The International Committee of Medical Journal Editors (ICMJE) and other organizations [8, 9] published a list of criteria for authorship requiring authors to: (1) design experiments, or analyse data, or interpret data; (2) draft or revise the manuscript; (3) approve the final manuscript; and, (4) agree to be held accountable for it [10, 11].

In addition to being accountable for the parts of the work they have done, authors should be able to identify which co-authors are responsible for other parts of the work.

Furthermore, they stipulate that any individual that contributed to the first activity be given the opportunity to participate in activities 2, 3, and 4, so as not to exclude them from authorship. The WAME criteria [8] are similar but somewhat broader and explicitly state that it is dishonest to disregard individuals that contribute to writing (ghost authorship) particularly those working for commercial companies. Like ICMJE they identify activities that warrant an acknowledgment:

Performing technical services, translating text, identifying patients for study, supplying materials, and providing funding or administrative oversight over facilities where the work was done are not, in themselves, sufficient for authorship, although these contributions may be acknowledged in the manuscript, as described below. It is dishonest to include authors only because of their reputation, position of authority, or friendship (guest authorship).

Whereas the IJCME require all authors to be familiar with all aspects of the work and capable of identifying who did what, the WAME criteria recognize that a biostatistician contributes complementary expertise and may be incapable of defending all the clinical aspects of the work. Harvard Medical School adapt these criteria and insist that specialized personnel be included even when their contribution is limited in scope. One individual of the team takes responsibility for the document and keeps a record of how everyone contributed [12], like the WAME guarantor or the corresponding author (CA) for the ICMJE.

The National Institutes of Health (NIH) expanded the ICMJE criteria and defined 15 activities related to publishing research [4, 13]. They discourage honorary authorship and recommend: excluding individuals that train/educate, provide resources, or read/comment the manuscript; always including researchers that draft the manuscript or perform original experimental work; and include individuals that participate in the other activities depending on their implication. Earlier critiques of the ICMJE guidelines alleged that they created orphan papers in which nobody met all 4 criteria and thus no one was eligible to be considered as an author [14] but this oversight has been corrected.

Authorship criteria aim to reduce unethical practices—coercive authors, honorary authorship, guest authorship, gift authorship and ghost authorship. A cross-sectional survey of corresponding authors publishing in 6 high impact biomedical journals confirmed the guidelines reduced ambiguous authorship from 29% in 1996 to 21% in 2008 but it was mostly due to reducing ghost authorship [5]. The survey identified 17 functions related to developing an article and asked how many co-authors contributed to only one, which would make them ineligible for authorship according to ICMJE [15]. Compliance to these criteria in ecological research is much lower [16] where 78% of the studies had at least one co-author that failed to meet ICMJE guidelines. Another study showed that in the top 1% of the highest cited articles across 22 Web of Science Core Collection (WoS) journal fields, one-third of them included a specialized author and one-half with non-author collaborators (ghost authors) [17].

Researchers disregard the guidelines because they are too restrictive as they discount the minutiae of research and the *plurality of value and the messiness of scientific practice* [18]. Moffat (2018) [19] argues that a universal consensus of assigning authorship is neither attainable nor desirable because it infringes on the autonomy of researchers scientific expression. Knowing whose suggestion, insight, or initiative contributed (and by how much) to research success is unknowable *a priori* and even *a posteriori*. Furthermore, comprehensive criteria must recognize that theoreticians value knowledge and writing as core activities while applied scientists and engineers value data reduction, maintaining, designing, and operating equipment and other tools more. Consider *Big Science* that tackles challenges facing society with hundreds and thousands of researchers working as a group. Experimental high energy physics articles approach 3000 individuals routinely [20, 21] and the record for the most authors is 5154 [22]. One quarter of the top 500 cited articles in nuclear physics averaged 1160 authors (WoS, 2010 to 2015) [23]. Author counts biomedical journals are not so high but 19 of the 244 articles *Lancet* published in 2017 had more than 40 authors, 10 had more than 480 authors, and one had 1039 [24].

## Materials and methods

We expanded the NIH authorship activity list and taxonomy classes of Allen et al., (2014) [25] to include 25 research tasks and developed a questionnaire to gauge the practices of researchers across all scientific endeavors. NIH list was specific to health and excluded activities often relegated to acknowledgments: peer interactive communications—advice, discussion, critical comment, and inspiration—[26], and access to experimental data, specimens (and equipment), technical or statistical help, editorial assistance, data gathering/data entry, and financial or moral support [27]. Our expanded list includes 5 classes with 5 activities per class to be able to compare quantitatively how people value each class. Any of these activities represents a substantial investment in time (a week and more) but not for all articles nor for all individuals.

We first developed the survey in Excel for a conference and refined the questions after feedback from students and colleagues [28]. In the following three months, we sent emails with a link to our refined questionnaire with the MonkeySurvey platform to students and staff at Polytechnique Montréal, colleagues from other institutes, and companies. We then posted the link on Facebook and LinkedIn. Approximately 400 people responded, most of whom worked in chemical sciences in Iran, Canada, and Italy. Half of these respondents were senior professionals/professors and the rest were graduate students, researchers with less than 5 years experience, and business people. Our next mailing list included researchers in various scientific fields from 15 institutes in the United States, Great Britain, France, Germany, Singapore, and Japan. Approximately 60 individuals completed the survey: 30 in the first mailing and another 30 after a reminder. We revised the questionnaire a final time stating that N/A (not applicable) was the same as not responding.

In October 2017 we sent emails to corresponding authors of the top 500 cited articles from 2010 to 2014 in 235 WoS categories [23]. The email mentioned the title of the CA’s paper, its rank within the scientific category, and the total number of papers in the category. It stated that the survey took 5 minutes to complete, had 5 categories with 5 questions each. Approximately 84 000 researchers received the email and 3500 responded while 30 000 were returned undelivered. Almost 12000 articles had no email address. WoS assigned 23000 to more than one category, and 21000 authors had two or more articles in the top 500.

A follow-up email included a link to bibliometric data of the 500 top cited articles in the CA’s scientific category. A further 3000 researchers attempted the survey at a completion rate of 91%. The overall response rate was almost 10%, which is less than half of the respondents in an earlier study related to contribution statements [29].

### Ethics statement

We stated the following in every email: *The online survey relies on a proprietary data collection system hosted on secure servers that ensures privacy and security. The data will gauge how you rank the importance of each research activity. We will publish this data in a peer reviewed journal. All data you contribute is strictly confidential and we commit to protect your anonymity in all reports and publications*. Polytechnique Montréal’s ethics committee (Comité d’éthique de la recherche avec des êtres humains) approved the study and we completed it according to the guidelines in CÉR-1617-21.

## SurveyMonkey questionnaire

The email we sent encouraging researchers to participate described the survey thusly:

*Hello*,

*Web of Science has indexed your paper entitled “Paper title” in the category “WoS category” and it was ranked “rank” out of “total number of articles” (published between 2010 to 2014). Was it easy for you to decide who deserved to be an author? Our survey of the top scientists in the world examines how they assign authorship*.

https://www.surveymonkey.com/r/GSPATIENCE2017

*So far, this survey demonstrates that we have diverse opinions. You will be surprised how much they deviate from criteria established by IJCME (International Committee of Medical Journal Editors)*.

*The survey takes 5 min and has 5 categories with 5 questions each*:

*Supervision*;

*Experimental design*;

*Generating, manipulating, analyzing samples*;

*Interpreting data; and*,

*Preparing manuscripts*.

*The IJCME criteria require that authors approve the manuscript and agree to be held accountable for it. Together with these two criteria, how often do you feel that any one of the 25 questions in the survey constitute sufficient contribution to merit authorship: almost always, usually, often, sometimes, or almost never*.

(see for more info http://onlinelibrary.wiley.com/doi/10.1002/cjce.22479/full).

*Skip any question that does not apply to your field*.

*Sincerely*,

Gregory S. Patience

Polytechnique Montréal

Telephone number

Ethics statement

In a follow up email, we summarized the country of origins of 3000 participants and shared the bibliometric data for the category in which WoS indexed their paper.

## Scoring

The five groups of questions resemble the classes of activities in journal contribution statements [29] with five activities per category. We corresponded with dozens of researchers to confirm that the questions targeted their perceived importance of each activity and not whether others or they themselves followed it in reality. We assigned a score *ω*_*k*_ to each response:

*ω*_0_ = 0: almost never—< 5% of the time;
*ω*_1_ = 1: sometimes—25% of the time;
*ω*_2_ = 2: often—50% of the time;
*ω*_3_ = 3: usually—75% of the time; and,
*ω*_4_ = 4: almost always—95% of the time.

This scoring scheme resembles a statistical distribution where *ω*_2_ = 2 represents the mean; *ω*_4_ = 4 represents the 95% confidence level and is 2 *σ* greater than the mean; *ω*_0_ = 0 is at the 5% confidence level, so it is 2 *σ* less than the mean; and finally, *ω*_3_ loosely represents 1 *σ* greater than the mean and *ω*_1_ represents 1 *σ* less than the mean.

The score, *s*_*i*_, of activity *i*, corresponds to the quotient of the sum of the responses and the total number of respondents: $s_{i}=\Sigma_{1}^{n_{i}}\omega_{i,k}/n_{i}$. When *s*_*i*_ > 3, the overwhelming consensus confirms that activity *i* merits authorship. When *s*_*i*_ < 1, most people think that the activity *i* seldom if ever, merits authorship. However, even for the activities where *s*_*i*_ < 1, over 1000 individuals chose usually or almost always. The aggregate individual score (index *j*) *S*_*j*_ = Σ_*q* = 1,25_*ω*_*jq*_ varies from 0 to 100 and we use this metric to compare responses across fields, geography, experience, and work place.

### Supervision

Supervision includes preparing grants, mentoring subordinates, and securing funding but does the time dedicated to these activities constitute sufficient intellectual involvement to be considered an author? Together with approving the final manuscript, any one of the five activities merits authorship: almost never (< 5% of the time), sometimes (25%), often (50%), usually (75%), or almost always (> 95%).

**Q1** Securing funding
**Q2** Establishing the team
**Q3** Coordinating tests
**Q4** Proposing ideas
**Q5** Providing resources (laboratory space, analytical, time)

### Experimental design (equipment)

Designing, operating, and maintaining experimental equipment are essential to generate data. Together with approving the final manuscript, any one of the five activities merits authorship: almost never (< 5% of the time), sometimes (25%), often (50%), usually (75%), or almost always (> 95%).

**Q6** Setting up experimental equipment, writing programs
**Q7** Designing equipment, writing programs
**Q8** Operating instruments and equipment, running programs
**Q9** Modifying, maintaining equipment and programs
**Q10** Troubleshooting mechanical failures

### Sample manipulation

Researchers generate, analyze and share samples with others for further analysis. Together with approving the final manuscript, any one of the five activities merits authorship: almost never (< 5% of the time), sometimes (25%), often (50%), usually (75%), or almost always (> 95%).

**Q11** Identifying necessary samples for the program
**Q12** Generating samples for external analysis (by collaborators or third parties)
**Q13** Supplying samples, computer programs
**Q14** Analysis of samples by third parties (that you pay for)
**Q15** Discuss results of samples, viability, reliability, error

### Data reduction

Together with approving the final manuscript, any one of the five activities related to manipuLating/analyzing data merits authorship: almost never (< 5% of the time), sometimes (25%), often (50%), usually (75%), or almost always (> 95%).

**Q16** Developing an experimental plan (DOE)
**Q17** Collecting/measuring experimental data, executing programs
**Q18** Consolidating experimental data
**Q19** Analyzing data, identifying trends
**Q20** Interpreting results, modelling, deriving correlations

### Writing

Writing papers includes adding text to sections, revising the document and responding to referees. Together with approving the final manuscript, any one of the five activities merits authorship: almost never (< 5% of the time), sometimes (25%), often (50%), usually (75%), or almost always (> 95%).

**Q21** Major role in drafting document
**Q22** Commenting on scientific content
**Q23** Correcting language, grammar, sentence structure
**Q24** Proofreading and suggesting substantial modifications
**Q25** Responding to reviewers/editors comments

**Fig 1:**
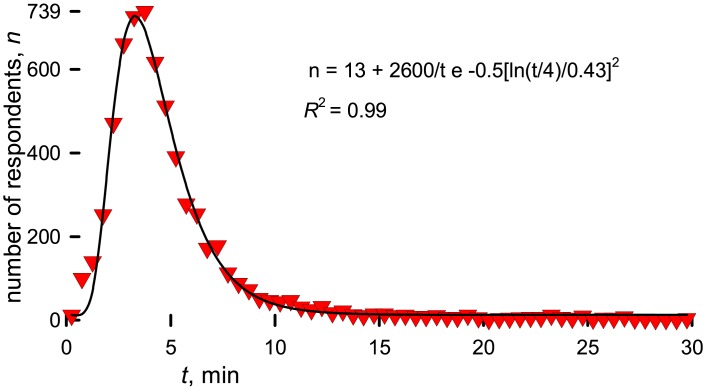
Time to complete the survey.

The questions remained succinct so as not to unduly influence the respondents and to minimize the time to do the survey. The mean response time was 5-minutes with a median of 4-minutes (Fig 1). Some commented that the questions were vague and thus open to interpretation but the email included references to an earlier study [4] with the NIH classification [13] and our contact information. Many referred to the article, few called, but we responded to 550 messages.

**Fig 2:**
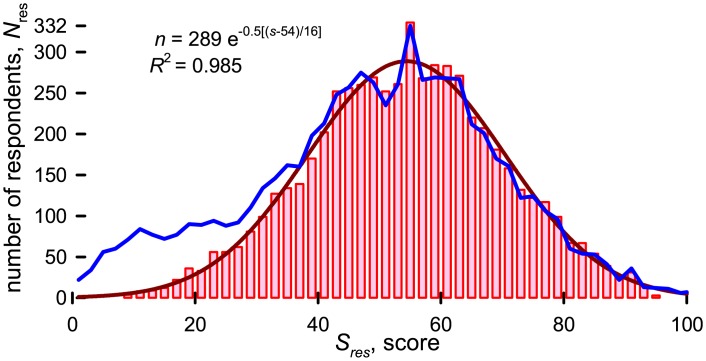
**Frequency of individual scores:** Blue line includes all responses, *N* = 6604, and an average score, *S*, of 48.6; bars represent retained responses, *N* = 5781 and an average score of 53.7; red line is the best fit Gaussian distribution, *R*^2^ = 0.985.

We retained 5781 responses of 6604 researchers that participated in the survey. We rejected responses that took less than one-minute to complete and those with a standard deviation equal to zero, which means all questions had the same response. We examined responses for which *S*_*j*_ > 90 and *S*_*j*_ < 10 and eliminated those whose responses were incomplete (*n* < 10) or incoherent. The blue line in Fig 2 represents the the scores of all individuals and the bars correspond to the retained responses. The standard deviation and mean for the reduced data set were 16 and 54.4, respectively, while for the entire data set they were 19 and 51.8.

Together with 25 questions related to research activities, we collected biographical data: seniority, country of birth, work place, and research discipline.

### Q26 Identify your level of experience

Senior researcher/professor (> 5 y)
Early career professional/professor (< 5 y)
Senior graduate student (> 2 y)
Junior graduate student (< 2 y)

Age is a factor related to research activities, with junior researchers (<6.8 y since the first published article) more likely to run experiments, while those with 10y experience analyze data, and senior researchers (<12 y experience) write, conceive experiments, and provide reagents [30]. Most of the respondents had at least 5 years of professional experience (5129), followed by early career professionals with less than 5 years (467). We considered that 5 years after a PhD, researchers will have had sufficient time and experience to form a measured opinion on research attribution, and this corresponds to about 12*y* experience. Few students participated in the study (180 with more than 2 years and 83 with less than 2 years) and even fewer in management (53).

### Q27 What is your country of birth?

Researchers born in 115 countries participated in the survey. Americans responded most (1296), followed by the British (452), Italians (369), Germans (359), and Canadians (339). The number of respondents from each country correlates reasonably well with the number of corresponding authors that wrote the most cited articles. Americans and British researchers were cited most and they responded most. Chinese authors ranked 3rd among the top cited articles but were ranked 7th in terms of responding to the survey. Both Japanese and Swiss researchers were among the top 10 in citations but were ranked 11th and 12th in number of responses. We identified the country of origin of 71474 corresponding authors; 11221 email addresses ended in .com (7871), .org (2892), and .net (458), while 82 came from 12 nations.

### Q28 Where do you work?

University
Government
Institution
Company
Other (please specify)

Most of the participants worked for universities (4214), followed by institutions (independent research institutes, non-governmental organizations, clinics, hospitals not associated with universities) (670), government (281), and companies (209). There were 206 individuals that chose *other* including: multiple affiliations—academic and hospital (47), private-practice and consultants (30), hospitals, international, and national institutes (49), non-governmental agencies and museums (20).

### Q29 What is your research discipline?

Over 1000 individuals cited multiple research disciplines like chemistry and physics, or behavioral neuroscience and psychology. Others referred to topics like molecular evolution, ultrasound, biomedical, and bone rather than specifying a category. We consolidated the answers and assigned each response to one of 21 scientific categories following closely the classification of Clarivate Analytics Science Watch [31] (Table 1). A total of 5500 responded to this question but over 1000 included multiple responses so the total was 6703.

**Table 1:** Participation rates for each category *j*, with a total number of participants *N*_*j*_, and total score, *S*_*j*_ as a function of scientific field and category. We grouped the 250 WoS sub-categories into 21 categories and then combined these into 4 **fields** based on the total score (*S*_*j*_) and a *t*-test comparing *s*_*i*_.

Field/Category	*S*_*j*_	*N*_*j*_	Field/Category	*S*_*j*_	*N*_*j*_
**Pure & Applied Sciences**	**59**	**1478**	**Humanities**	**47**	**1312**
Material Science	61	170	Mathematics	49	69
Physics	59	237	Literature	48	14
Chemistry	58	400	Health Science	48	157
Engineering	58	671	Education	47	94
			Economy/Business	47	358
**Natural Sciences**	**54**	**2455**	Psychology	46	414
Agriculture	55	53	Sociology	46	206
Biology	55	842			
Medicine	55	723	**Philosophy, Law, Political Science**	**40**	**149**
Computer Science	54	222			
Statistics	54	22	Political Science/Law	43	111
Archaeology/Anthropology	54	84	Philosophy	38	38
Geosciences	54	388			
Environmental Science	53	121			

The lowest numbers of respondents were in literature (14), statistics (22), and philosophy (38). Researchers in the humanities, mathematics, theoretical physics, and economics stated that most of the questions did not apply to them. Individuals in biology, medicine, engineering, and chemistry responded with the highest frequency, which corresponds to the most categories in WoS (Table 1).

## Results

**Fig 3:**
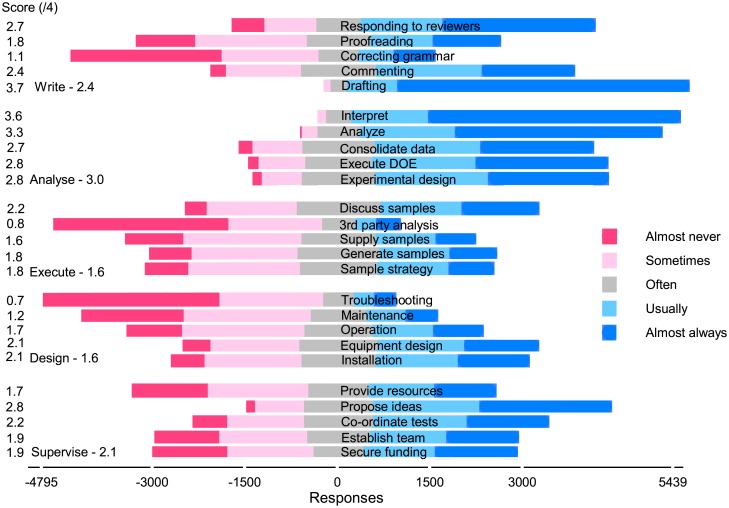
All fields Likert chart: 5500 respondents. The number of responses less than zero on the *x*-axis include almost never (*ω*_0_), sometimes (*ω*_1_) and half of the often (*ω*_2_) responses. The number of responses greater than zero include almost always (*ω*_4_), usually (*ω*_3_), and half of the often responses. The mean scores on the *y*-axis are $s_{i}=\Sigma_{1}^{n_{i}}\omega_{i,k}/n_{i}$ and varies from a low of 0.7 (troubleshooting) to a high of 3.7 (drafting).

Most CAs agreed to assign authorship to those that drafted the manuscript (*s*_21_ = 3.7), interpreted data (*s*_20_ = 3.6), and analyzed data (*s*_19_ = 3.3), which agrees with the ICMJE criteria (Fig 3). However, unlike the ICMJE, they attributed authorship to many other activities like proposing ideas (*s*_4_ = 2.8), consolidating data (*s*_18_ = 2.7), executing a DOE (*s*_17_ = 2.8), experimental design (*s*_17_ = 2.8), and responding to reviewers (*s*_25_ = 2.8). Execute (sample management) and design (including operation) were the least valued activity classes, but even so, thousands of people thought that activities in these classes always merited authorship.

Researchers were most ambivalent about supervision: with an score of the five activities of 2.1: more than one thousand thought these activities almost always merit authorship and about the same thought they almost never merit authorship. Proposing ideas scored 2.8 (${\bar{s}}_{4}$), ranking it fourth among the 25 activities, while providing resources scored poorly at 1.7 (${\bar{s}}_{5}$). Even so, one third of the respondents indicated that providing resources merits authorship almost always or usually.

**Fig 4:**
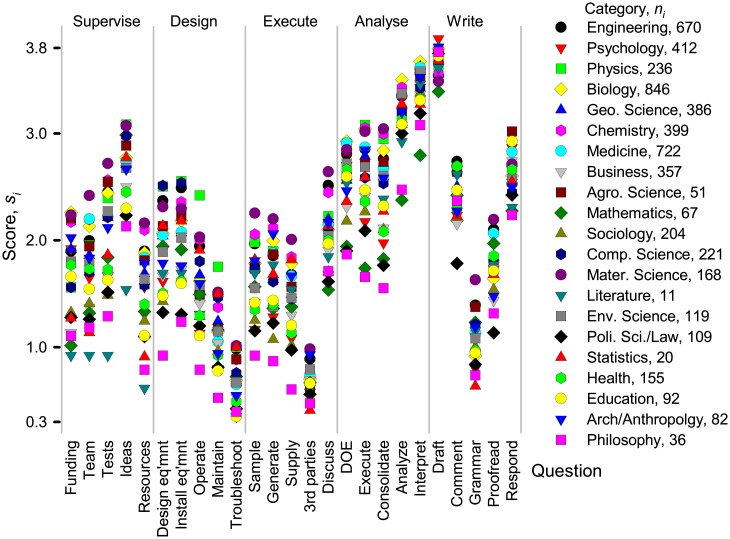
Mean scores, *s*_*i*_, according to scientific category. We grouped 5363 respondents according to the Clarivate Analytics science category definitions [31] and then calculated a mean score per question for each category based on the total number of respondents per category, *n*_*i*_: $s_{i}=\Sigma_{1}^{n_{i}}\omega_{ik}/n_{i}$.

The responses ranged from almost always to almost never for most activities independent of the scientific category, even for people in the same region, and same level of experience. However, to identify differences in general tendencies, we calculated mean scores according to category (Fig 4). The standard deviation between category and means were highest for drafting the document and analyzing data. Scores diverged substantially for the other activities, although the trends were similar. The lowest response rates were from philosophy and literature and they also had the lowest scores across most questions: they were most unlikely to grant authorship for anything other than writing and analysis. Researchers in materials sciences were more likely to grant authorship for other activities.

**Fig 5:**
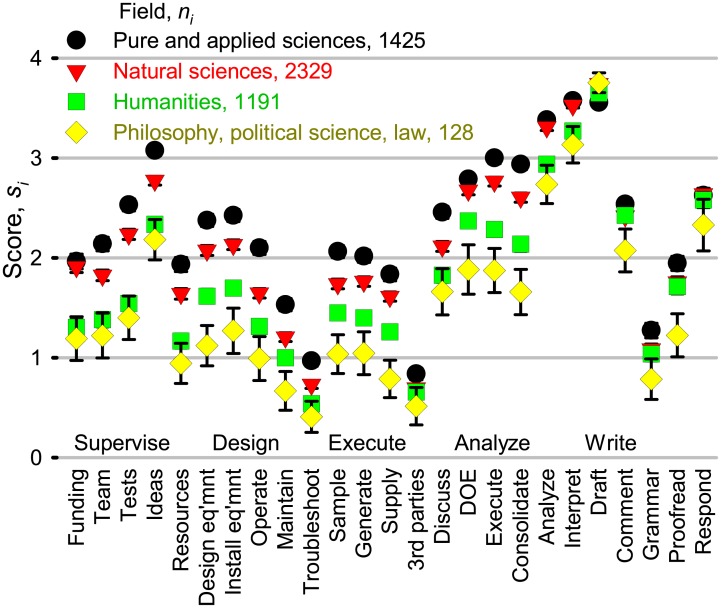
Mean scores according to scientific field. We compared the aggregate category scores, *S*, and assigned them to one of four fields based on the Student’s *t*-test. Pure and applied sciences includes physics, chemistry, materials, and engineering (*S* = 59). Natural sciences includes biology, geosciences, medicine, computer science, agricultural sciences, environment, statistics, and archeology (*S* = 54). Many humanities categories made up the third field (*S* = 48): psychology, management, mathematics, sociology, literature, education, and health. The final field (*S* = 41) comprised philosophy, political science, and law. The error bars depict 95% confidence intervals, *n*_*i*_ is the number of responses per category, and $s_{i}=\Sigma_{i}^{n_{i}}\omega_{ik}/n_{i}$. (Symbol sizes are larger than most error bars).

A bi-dimensional analysis clustered 1 009 411 acknowledgments from articles indexed by WoS from 2015 into six fields: biology, clinical medicine/health, biomedical research, social sciences, geosciences and space sciences, and chemistry, physics and engineering. Psychology spanned the last cluster and the clinical medicine/health cluster [32]. Based on the average score of each category, *S*_*j*_ and a paired *t*-test comparing the responses to the 25 questions, we grouped the categories into only four fields—pure and applied sciences, natural science, humanities, and philosophy, law, and political science (Fig 5). Both acknowledgment and contribution statements identify differences in collaboration patterns across disciplines. However, our survey clearly places psychology together with humanities rather than with pure and applied sciences. Moreover, geosciences belongs with natural sciences rather than humanities.

The highest average score was for the pure and applied sciences at *S* = 59 and it was lowest for philosophy, political science at *S* = 41. Differences between pure and applied sciences and natural sciences (*S* = 54) were about the same as that between natural sciences and humanities (*S* = 48). The size and scope of collaborative teams in the humanities is much lower than in pure and applied and natural sciences, which accounts for some of the difference in the scores [23].

**Fig 6:**
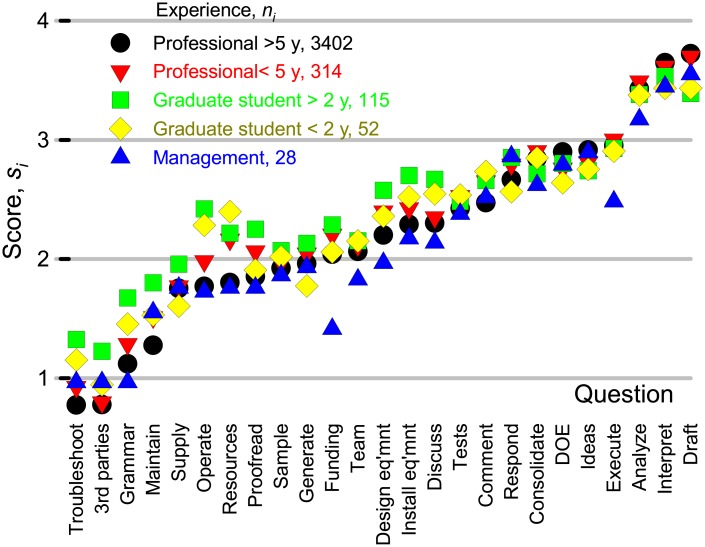
Scores for each of the questions with respect to career experience. The data set includes responses from pure and applied sciences and natural sciences and excludes those from Asia and Africa. The questions were organized from lowest to highest score based on the answers of the professionals with 5 years of experience where *n*_*i*_ is the number of responses for each of the 4 categories of experience and $s_{i}=\Sigma_{i}^{n_{i}}\omega_{ik}/n_{i}$.

To test whether opinions varied according to experience and birth country, we only considered pure and applied and natural sciences (column 1 of Table 1). We ordered aggregate scores from each question, *s*_*i*_ from the lowest to the highest(Fig 6). Responses of professionals with less than 5 years of experience and those with more than 5 years were highly correlated (*R*^2^ = 0.990). Graduate students (predominantly Master’s) had the second highest correlation with the professionals (*R*^2^ = 0.95). Graduate students with more than 2 years of experience viewed the contributions from each of the activities more favorably, while business people were less inclined to grant authorship to most of the activities. For all groups, the highest three and lowest three ranked activities were the same. Most thought that preparing a grant proposal would often merit authorship while business people thought that it would warrant authorship only sometimes.

To test tendencies based on linguistic and regional circumstances, we initially grouped the countries into 8 categories: (1) hispanophone (Portuguese + Spanish), (2) anglophone (including Canada with a significant French population), (3) Eastern European (including ex-USSR states), (4) South Asia (including Iran, Afghanistan, and Pakistan), (5) Western Europe (and Israel), (6) sub-Saharan Africa, (7) Middle East and North Africa, (8) and Far East Asia. We then compared the means of responses from individual nations against the regions. Based on a *t*-test comparing the *s*_*i*_ of each country against the group average, we reformed the groups. Responses from Italians, French, Greek, and Cypriots resembled the hispanophones more than the rest of Europe, so we grouped them together and labeled them Latin (although Greek is not a Latin language). Northern Europe we labeled Germanic even though Finnish is not Germanic. We also labeled former Eastern European countries Slavic although Hungary, Romanian, and some of the Soviet Bloc states speak other languages. Because the sub-Saharan Africa group only had 22 respondents, we combined it with the Middle East and North Africa (MEA). We could have grouped these nations with anglophones as most are part of the Commonwealth. Based on the original *t*-test, Iran belonged with South Asia but with the expanded MEA grouping, it belongs to either South Asia or MEA, so we regrouped Iran with the latter.

**Fig 7:**
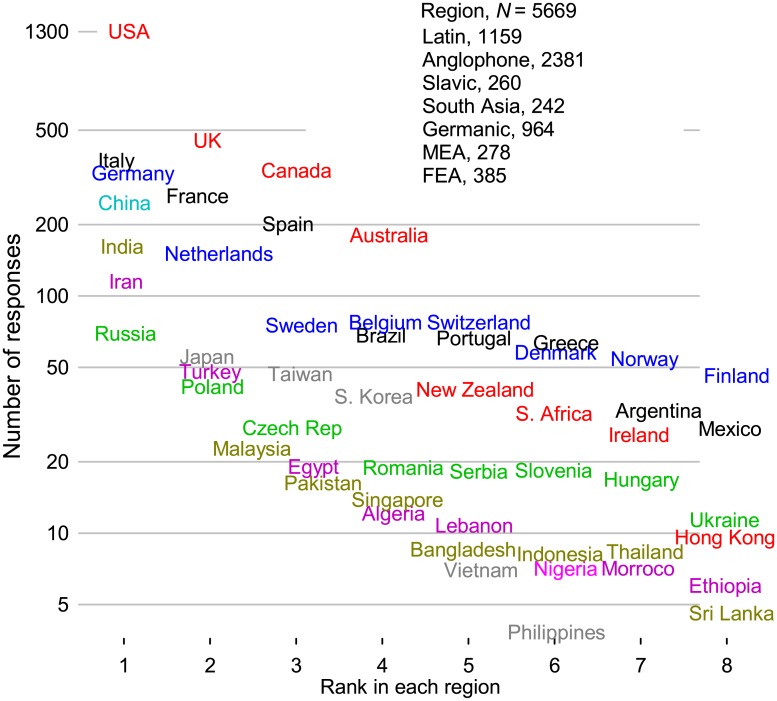
Number of respondents from each country and grouped in regions.

The sample size for answers to countries was 5669 (Fig 7). Americans responded most and the anglophone group had twice as many respondents as the Latin and Germanic groups. Germany followed by the Netherlands headed the Germanic group and the next 5 countries each had at least 50 respondents. The distribution for then number of respondents per country for the Latin group was similar with Italy heading, followed by France and Spain. The other regions had a similar representation that varied from 242 (South Asia) to 385 (Far East Asia).

**Fig 8:**
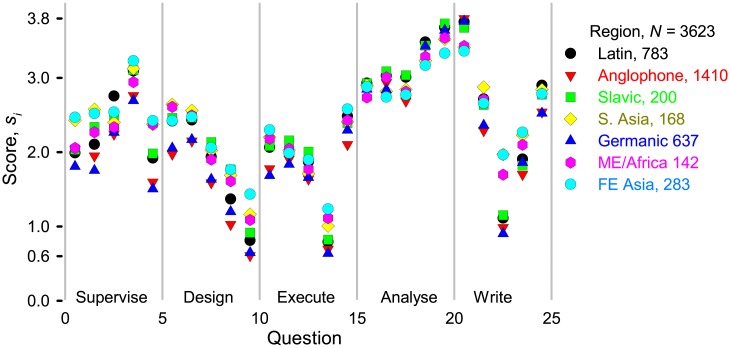
Activity scores as a function of region. Researchers from 115 countries answered the survey and we grouped them into regions based on *t*-tests comparing the mean score ($\bar{S}$) of the country and region. The Latin region includes South American countries, Portugal, Spain, Italy, France, Greece, and Cyprus. Anglophones include the United States and many Commonwealth countries. Slavic nations comprise Eastern Europe and former Soviet states. India, Pakistan, Indonesia, Singapore, and Bangladesh are part of the South Asia region. The Germanic states include European countries north of France/Italy. Middle East and Africa (MEA) is the most diverse grouping and includes Iran, Turkey, Arabic-speaking nations and sub-Saharan Africa (some of which could have been included among the Commonwealth nations). China, Japan, South Korea, Taiwan, Vietnam comprise the Far East Asia region (FEA).

The differences between regions were much less than the differences between categories (Fig 8) and so birth place is an inconsequential contributor to the huge variance shown in Fig 3. Some slight differences are evident: anglophones responded similarly to northern Europe (Germanic) each with *S*_*j*_ = 53, which means they are less generous assigning authorship than the other regions. Researchers from Far East Asia (FEA) and south Asia recognized non-standard contributions (that is, supervise, design, and execute) most (*S*_*j*_ = 62 and 61), while anglophones and the Germanic cluster recognized these least. Excluding sub-Saharan Africa and Iran, *S*_*j*_ = 62 for MEA. According to a *t*-test of the means, the responses from Israel were indistinguishable from the Germanic region. The responses from the Slavic group were much like the Latin group at *S*_*j*_ = 59. Correcting grammar is among the least valued activities but FEA and MEA researchers rank it close to a full point higher than researchers from other regions.

## Discussion

For almost every category, opinions range between the two extremes (Fig 3): as many people think that they should rarely ever merit authorship as those that think is should almost always merit it. Describing the data statistically and comparing responses across fields, countries, and profession identifies trends but the large variance show how divergent opinions are. Even within the same discipline, same region, and same level of experience, responses extended from one extreme to the other. For all responses except *responding to reviewer’s comments* (Q25), *p* < 0.05 for a two-tailed *t*-test comparing pure and applied sciences versus philosophy, political science, and literature (Fig 5). The responses from scholars in the humanities and in philosophy were generally indistinguishable—*p* < 0.01 for questions 4, 5, 6, 13 16, and 24. The *p*-values for *t*-tests between the natural sciences and pure and applied sciences were greater than 0.05 for questions 2, 16, 19, 20 and 25.

**Fig 9:**
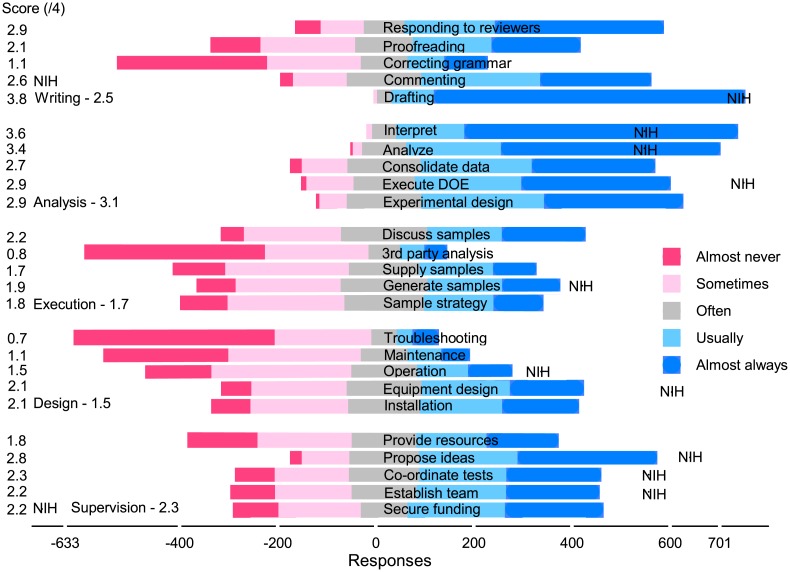
**Medical sciences Likert chart:** 720 respondents. The number of responses less than zero on the *x*-axis include almost never (*ω*_0_), sometimes (*ω*_1_) and half of the often (*ω*_2_) responses. The number of responses greater than zero include almost always (*ω*_4_), usually (*ω*_3_), and half of the often responses. The mean scores on the *y*-axis are $s_{i}=\Sigma_{1}^{n_{i}}\omega_{i,k}/n_{i}$. *s*_*i*_ varies from a low of 0.7 (troubleshooting) to a high of 3.8 (drafting).

The top cited authors disregard the ICJME criteria including 722 researchers in medicine for whom the criteria were created 30 year ago (Fig 9): they continue to recognize contributions other than those outlined by the IJCME. The NIH rated how often activities in their guidelines merited authorship and we have reproduced their estimates in Fig 9 (NIH label). Their scores agree with the respondents except for commenting on the manuscript and providing resources with scores of 2.6 (*s*_22_) and 2.2 (*s*_1_), respectively.

Individuals in the humanities, mathematics and theoretical physics, stated that collaborations were less common compared to other disciplines and that many of the questions did not apply, particularly those pertaining to experimental equipment. As a result of the smaller research teams and presumably lower multidisciplinarity, the respondents were less likely to attribute authorship to anything but writing, responding to reviewers, and analysis. Studies on contribution statements confirm this tendency [30]. Philosophers, politicians and lawyers rank contributions from activities other than writing lower than all other categories (Fig 5). Humanities share their opinion for supervision but value the other categories slightly more but much less than natural sciences and pure and applied sciences. For philosophers, the reasoning process to support an idea or theory represents the essential element of research. Supervision, funding, infrastructure, and editorial contributions, are less valued. Experimental physics is on the other end of the research spectrum in which thousands of physicists, engineers, and other scientists work together in teams with time-lines that span decades, like for the Large Hadron Collider at CERN. Clinicians conduct cross-institutional research and must build international relationships to foster goodwill to plan for future collaborations with groups who have access to patients and data, for example [33]. Because of the chasm between physical experimentation and philosophical argumentation, some respondents recommended independent surveys that target specific activities for groups of categories.

Research complexity and funding promote collaborations and so categories with larger teams and infrastructure recognize a broader range of contributions for authorship. Anglophones recognize editorial contributions less than individuals whose mother tongue is something other than English. Furthermore, writing English is more difficult for Far East Asians than for Germans or Indo-Europeans. For this reason, Chinese and Japanese researchers attribute authorship for editorial contributions more readily (Fig 8).

We were surprised that the survey identified tendencies related to geography, language, and historical relationships. South Americans have a historical affinity with Spain and Portugal and they assigned authorship like Italians and Greeks. According to the survey, French scholars assign authorship more like this group rather than the Anglo-Germanic groups, and, coincidentally, their cultures and languages are more closely related. Although Iran is considered a Middle Eastern country, Iranians recognize authorship like Southern Asians, which suggests that they have a stronger cultural affinity. Israel belongs to the Middle East geographically, but their research culture originates from northern Europe and thus Israelis attribute authorship like the Anglo-Germanic group. This survey definitely identifies culture as a factor assigning authorship.

The ICMJE and NIH recommend how to attribute authorship but our survey demonstrates that the most successful researchers in the world recognize intellectual content beyond their criteria—research is messy and universal guidelines may not infringe on the autonomy of scientific practice [19, 34]. Assigning authorship relies on fairness on the part of principal investigators (PI) that receive public and private funding. They have a duty to conduct research responsibly, which comprises honesty, integrity, openness, and transparency. PIs have the authority to choose individuals and groups to conduct the work, the authors, and author order. However, PIs have an obligation to share their authorship criteria so that everyone understands how they will be recognized—authorship, acknowledgment, financial compensation, etc. A point system that assigns weights to research activities is one approach to evaluate contribution [35, 36]. The PI assesses input from each of the contributors and divides points of each activity among them. Individuals can contribute to several activities and anyone that exceeds a predetermined threshold becomes an author. Several respondents to the survey argued that contributing to only one activity may be insufficient to merit authorship but contributing to many activities could be sufficient.

Tangible research outputs include ideas, data, designs, writing, graphs, programs, and methodologies and these may be protected by copyright or patents. Researchers have the sole right to reproduce, distribute, publish, and create derivatives of their original work. As soon as work is recorded (written), publicly or privately, it is covered by copyright, which raises questions regarding how to acknowledge professional writers, particularly those with subject specific knowledge that correct and edit not only text but also improve scientific content [37]. Excluding creators of original work constitutes copyright infringement, regardless of how few points they have accrued. Copyright does not protect ideas but copying an idea, data (interpretation), methodologies could be plagiarism. The system of points is appropriate to assign author order but other considerations may trump it for attributing authorship.

## Limitations

We sent the survey to the top corresponding authors indexed by WoS, which introduces a bias towards experienced senior researchers. These individuals have the authority to choose collaborators and decide who merits authorship. However, several hundred junior researchers did respond and many professors shared the survey with their students, thus we had some responses to gauge their opinions.

The survey has four biographical questions and we assumed where people were born (culture) influences their attitudes more than where they completed their doctoral or post-doctoral studies. However, the corresponding authors country of origin resembled the respondents’ country of origin with respect to percentage and rank. More questions would have made it clear how many studied in the same country they were born but we minimized these types of questions. Canada, Italy, India, and Iran were over represented in the survey versus the number of citations because of the author’s ties with these countries. Three individuals chose Europe as their birth country, while 5 questioned the relevancy of the question. One person replied *No way* while another stated *This totally misses the fact that many scientists do not work where they are born. I do not see the point of this question otherwise*.

We corresponded with over 550 individuals and exchanged 1520 email: 104 thought the work was very important or wished us luck; 84 thanked us for the bibliometric information concerning their work and for the survey; 93 asked us for more information; 85 made comments and confirmed that they completed the study; 59 said the survey did not apply to their discipline or that it was irrelevant as they were the sole author of the paper (mostly mathematicians, theoretical physicists, and social sciences/humanities); 53 had problems with the Survey Monkey website; 40 thought the survey was unclear; 21 wished to unsubscribe; and, 20 asked if it was a spam.

For researchers who were sole authors or who said that it was irrelevant, we asked if they worked with students, wrote grant proposals, collaborate with colleagues, or analyzed data that they themselves did not collect. Faced with this additional input, some accepted to complete the survey. The most frequent question was if they were to respond with respect to what they did, what they thought they should do, or what the others did in the field. For those who were sole authors, we asked them to consider how they thought people in the field assigned authorship. Otherwise, we were expecting the researchers to respond based on how they now attribute authorship (which we would expect to be similar how they did it for their paper). Some refused to complete the questionnaire without this clarification. Others criticized the survey saying that it was very obscurely worded, hashed up and a bogus approach to research or that the questions were ambiguous. Researcher suggested that the diversity of opinion was in fact because research enquiry involves multiple tasks and the sum merits authorship. Examples would have helped to clarify what we meant by *samples* and *writing programs*.

## Conclusion

Scientists and engineers publish to build a reputation that universities, companies, and governmental agencies examine to hire, promote, and fund. Research complexity continues to increase requiring larger multi-disciplinary teams. Consequently, authorship lists are growing, so journals require corresponding authors to disclose everyones contribution to ensure equitable recognition—authorship or acknowledgment. However, corresponding authors continue to include individuals with a modicum of intellectual involvement [38] and to exclude those with substantial intellectual contribution. Consider hyper-prolific authors that publish an article every five days: despite their evident devotion, hard work, extensive collaborations, and organizational capacity, publishing a paper every 5 days would seem an inconceivable endeavor. [39].

Our survey has provoked broad interest [40] and demonstrates that people value activities beyond writing and analyzing data but the opinions are polarized: As many researchers credit activities like supervision almost always as those that credit them almost never, even people in the same field and country. Pure and applied scientists grant authorship outside the ICMJE guidelines more compared to those in the humanities. This prevalence is related to large collaborations and the importance of building social relationships. Furthermore, adding individuals with imperceptible contributions is more tolerable than excluding those adding creative content.

The ICMJE guidelines provide researchers a means to discuss social pressures regarding authorship. *PNAS* organized an exclusive conference of individuals from among the largest scientific publishing houses and proposed stricter guidelines for authorship [41]. Our survey suggests that although many researchers are willing to follow more rigorous criteria, as many others will ignore them. The most egregious authorship practices researchers mention are those individuals that require authorship for *standard/unique* experimental tests. Individuals with unique infrastructure or access to limited resources chose the conditions of their collaboration—money or authorship. When they are not motivated by money, authorship is the only choice. On the other end of the spectrum, when scholars leave without completing the research or approving a draft, according to most guidelines, the work is unpublishable. Whatever the authorship criteria CAs adopt, to avoid conflict and potential disputes related to plagiarism, they must develop and share authorship criteria before engaging collaborators. They should also consider the culture of individuals with whom they collaborate to minimize potential conflicts.
